# Multi‐omics reveals a novel Cxcr4^+^ subpopulation of alveolar macrophages and therapeutic effect of AMD3100 in mice with advanced silicosis

**DOI:** 10.1002/ctm2.70705

**Published:** 2026-05-27

**Authors:** Min Mu, Bing Li, Hangbing Cao, Yuanjie Zou, Ruiqing Yan, Fei Wang, Yehong Zhao, Zihao Xie, Miaomiao Du, Xiaolong Wu, Xinrong Tao, Jianhua Wang

**Affiliations:** ^1^ School of Public Health Anhui University of Science and Technology Huainan China; ^2^ Key Laboratory of Industrial Dust Control and Occupational Health of the Ministry of Education Anhui University of Science and Technology Huainan China; ^3^ State Key Laboratory of Safe Mining of Deep Coal and Environmental Protection Huainan Mining (Group) Huainan China; ^4^ Medical school of Anhui University of Science and Technology Huainan China; ^5^ Anhui Shendong Biotechnology Development Co., Ltd. Huainan China; ^6^ Cancer Institute Fudan University Shanghai Cancer Center Fudan University Shanghai China

**Keywords:** alveolar macrophages, AMD3100, multi‐omics, silicosis

## Abstract

**Background:**

Silicosis is a work‐related condition resulting from breathing in crystalline silica particles, marked by persistent inflammation and abnormal healing mechanisms in the lungs. Our previous studies demonstrated that inhibition of Cxcr4 with Plerixafor (AMD3100) markedly attenuates pulmonary fibrosis.

**Methods:**

By integrating single‐cell RNA sequencing with spatial transcriptomics, we analysed lung tissues from a mouse model of pneumoconiosis. Using the Robust Cell Type Decomposition algorithm to deconvolve spatial transcriptomic data, we identified Cxcr4^+^ macrophages and Cxcl12^+^ fibroblasts as central drivers of pulmonary fibrosis progression, revealing a distinct spatial co‐localisation pattern between these cell populations. To further delineate macrophage heterogeneity and functional specialisation during silicosis progression, we focused on key macrophage subpopulations.

**Results:**

AMD3100 dynamically remodels the alveolar macrophages (AMs) niche, promoting the restoration of AM homeostasis and significantly reducing both co‐expression and spatial co‐localisation of Cxcr4/transforming growth factor‐β (TGF‐β) signalling within macrophages, thereby modulating the fibrotic immune microenvironment. Mechanistically, silica dust stimulation in vitro upregulates Cxcr4 expression in the AM cell line MH‐S, which in turn promotes the release of TGF‐β and pro‐inflammatory factors, driving fibroblast activation. Activated fibroblasts further enhance the pro‐fibrotic phenotype of macrophages via secretion of Cxcl12, reinforcing the Cxcr4 signalling axis and establishing a stable positive‐feedback loop.

**Conclusion:**

Our findings suggest that silicosis‐associated fibrosis progresses through a positive feedback loop involving interactions between Cxcr4^+^ AM macrophages and Cxcl12^+^ fibroblasts. These findings highlight the therapeutic promise of targeting the Cxcl12/Cxcr4 axis with AMD3100 as an innovative approach for silicosis treatment.

## Key points

1. Targeting Cxcr4 with AMD3100 alleviates silica‐induced pulmonary fibrosis, improves lung function, and restores the dysregulated cellular ecosystem in vivo.
2. Multi‐omics analyses reveal that the Cxcl12/Cxcr4 axis drives a pathogenic positive feedback loop between macrophages and fibroblasts in silicosis.
3. AMD3100 disrupts the macrophage‒fibroblast crosstalk by blocking the Cxcl12/Cxcr4/transforming growth factor‐β signalling axis, providing a mechanistic basis for therapeutic intervention.

## Graphical abstract

The progression of silicosis‐associated fibrosis relies on a positive feedback loop mediated by crosstalk between Cxcr4^+^ macrophages and Cxcl12^+^ fibroblasts and the therapeutic potential of targeting the Cxcl12/Cxcr4 axis with AMD3100 as a novel strategy for treating silicosis.

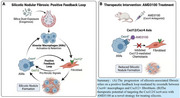

## 1 INTRODUCTION

Silicosis is an irreversible and often fatal fibrotic lung disease caused by the prolonged inhalation of crystalline silica (SiO_2_) particles, predominantly affecting workers in mining, construction and manufacturing industries.^1^ It remains a significant global health burden, particularly in developing countries where occupational exposure is poorly controlled, resulting in thousands of deaths annually despite the availability of known preventive measures.^2^, ^3^ The pathogenesis of silicosis is complex and multifactorial, with macrophages playing a central role in both the initiation and progression of the disease.

When inhaled, silica particles are engulfed by alveolar macrophages (AMs), causing lysosomal damage, the production of reactive oxygen species and the discharge of damage‐associated molecular patterns. This initiates a sequence of persistent inflammation and abnormal tissue healing mechanisms.^4^ This inflammatory milieu is further exacerbated through the activation of specific molecular pathways. Macrophages, however, are not merely inflammatory agents; they are master regulators of tissue homeostasis and repair. In silicosis, their reparative functions become profoundly dysregulated. Single‐cell RNA sequencing (scRNA‐seq) analyses have identified a distinct pro‐fibrotic macrophage subpopulation within fibrotic lesions, characterised by high expression of Spp1 and specific metabolic alterations that reinforce a pro‐fibrotic phenotype.^5^ These specialised macrophages, often referred to as Spp1hi macrophages, secrete potent fibrogenic mediators, including transforming growth factor‐β (TGF‐β) and tumour necrosis factor‐α, which directly activate resident lung fibroblasts, driving their differentiation into collagen‐producing myofibroblasts and leading to excessive extracellular matrix deposition.^6^ This macrophage‒fibroblast communication is sustained through multiple mechanisms, including the release of soluble factors, exosomes containing microRNAs and proteins, and potentially even direct cellular transformation, creating a self‐perpetuating cycle that underlies progressive pulmonary scarring.^7^, ^8^, ^9^ The resulting fibrotic tissue gradually replaces functional lung parenchyma, leading to restrictive lung disease, respiratory failure and ultimately death.

A growing body of evidence implicates the C‒X‒C motif ligand 12 (Cxcl12)/C‒X‒C chemokine receptor type 4 (Cxcr4) chemokine axis as a central regulator of fibrosis across various models, including silicosis.^10^ Upregulated in response to endoplasmic reticulum stress, hypoxia and inflammation, Cxcl12 binds to Cxcr4 to recruit fibrogenic monocytes and fibroblasts to injury sites, activate fibroblasts and synergise with TGF‐β signalling, forming a potent pro‐fibrotic feedback loop.^11^ Even in pancreatic ductal adenocarcinoma, Cxcr4^+^ macrophages have been shown to promote extracellular matrix remodelling, tumour proliferation and migration. In the context of pulmonary fibrosis, recent studies have demonstrated that inhibiting the Cxcl12/Cxcr4 axis ameliorates lung fibrosis. Mechanistically, fibroblast‐derived Cxcl12 acts via Cxcr4 to recruit fibroblasts and promote fibrotic progression. Additionally, Cxcr4 expression on epithelial cells has been identified as a potential therapeutic target, with similar findings reported in chronic obstructive pulmonary disease.^12^, ^13^, ^14^ However, none of these studies specifically examined the role of Cxcr4^+^ macrophages in fibrosis. Therefore, in the present study, we focused on investigating the Cxcl12/Cxcr4 signalling axis in silicosis and pulmonary fibrosis. While the therapeutic potential of Cxcr4 inhibition has been demonstrated, the precise cellular mechanisms by which this axis coordinates the pathogenic crosstalk between macrophages and fibroblasts in silicosis remains unclear. Current research indicates that in a mouse model of polymyxin‐induced pulmonary fibrosis, AMD3100 demonstrates marked anti‐fibrotic properties. Additionally, in the context of neuroinflammation, AMD3100 plays a significant role in mitigating neuroinflammatory responses.^15^, ^16^ These findings are consistent with our prior studies.

Here, we applied scRNA‐seq and spatial transcriptomics (ST) to delineate the cellular landscape of silicotic fibrosis. Our analysis revealed a high degree of spatial co‐localisation and intricate communication between macrophages and fibroblasts, with the Cxcl12/Cxcr4 axis emerging as a dominant signalling pathway. We identified a key subpopulation of Cxcr4^+^ macrophages recruited by fibroblast‐derived Cxcl12, which in turn drives fibroblast activation through TGF‐β, establishing a vicious cycle. Our findings elucidate how this localised interaction perpetuates disease progression and demonstrate that its therapeutic disruption via Cxcr4 inhibition effectively ameliorates the fibrotic process, revealing a promising avenue for clinical intervention.

## 2 MATERIALS AND METHODS

### 2.1 Animals and treatments

Male C57BL/6 mice (10‒12 weeks old) were purchased from Changzhou Kavion Company (license no. SCXY (Su) 20110003). The animals were housed in a regulated setting featuring a light/dark cycle lasting 12 h, a maintained temperature of 23 ± 1°C, 50% humidity and free access to food and water. All procedures involving animals were conducted in accordance with the NIH Guide for the Care and Use of Laboratory Animals and received approval from the Animal Ethics Committee at Anhui University of Science and Technology (approval no. 2021018).

After a 1‐week acclimatisation phase, the mice were randomly assigned to one of four experimental groups, with 11 animals in each group: the control (N) group, which was given intranasal instillation of phosphate‐buffered saline (PBS), and the silicosis (S) group, which received intranasal instillation of silica particles at a dose of 12 mg/60 µL.^17^, ^18^ Silicosis + AMD3100 (A) group: received silica instillation followed by AMD3100 treatment. AMD3100 (AMD) group: received PBS instillation followed by AMD3100 treatment. Crystalline silica (99% purity,  .5‒10 µm, ∼80% within 1–5 µm; Sigma‒Aldrich, S5631) was instilled intranasally according to a method previously established in our laboratory.^19^, ^20^

At 8 PM, the silicosis model was induced in the S and A groups by a single intranasal instillation of silica suspension. N and AMD groups received an equal volume of PBS via the same route. Starting 12 h post‐instillation, mice in the A and AMD groups received daily intraperitoneal injections of AMD3100 (5 mg/kg, i.p.; HY50912, MCE), while mice in the N and S groups received daily injections of an equivalent volume of PBS. This treatment regimen continued for 7 days.

On day 14, all mice underwent enucleation for blood collection, followed by euthanasia. Prior to lung tissue collection, animals received cardiac injection of PBS to clear circulating blood. Lung tissue was then harvested. The lung tissue was fixed in 4% paraformaldehyde for subsequent immunohistochemical analysis.

### 2.2 Single cell and spatial transcriptomics

Before proceeding with scRNA‐seq and ST analysis, the following is a description of the processing and sequencing preparation for whole‐lung tissue from mice.

#### 2.2.1 Single‐cell sequencing analysis

Lung tissues from mice were initially cut into fragments of roughly 2 mm^3^, cleaned twice with sterile PBS, and then promptly placed into a cold tissue preservation solution. Following the standard protocol of 10× Genomics, single‐cell suspensions were subsequently prepared. In summary, the tissues underwent enzymatic dissociation, and the resulting cell suspensions were filtered using a strainer to eliminate debris. Before being loaded onto the 10× Genomics Chromium platform, viable cell counts and concentrations were determined. The processes of single‐cell capture, barcoding and library construction utilised the 10× Genomics Chromium Single Cell 3′ Reagent Kit (version 3.1). Libraries were sequenced on an Illumina NovaSeq 6000 system, aiming for approximately 50 000 read pairs per cell. All steps, from the dissociation of tissues to sequencing, were carried out by OE Biotech Co., Ltd.

The analysis of the raw sequencing data was conducted with the Cell Ranger software suite tailored for single‐cell applications (version 7.0.1; 10× Genomics), which facilitated demultiplexing, barcode processing and alignment to the mm39 (GRCm39) reference genome of mice. Following this, bioinformatic analyses were conducted in R (version 4.0.3) utilising the Seurat package (version 4.3.0). Cells were filtered according to specific quality control parameters: detection of fewer than 200 genes per cell, total unique molecular identifier (UMI) counts being less than 1000, or a mitochondrial gene proportion greater than 5%. Potential doublets were detected and eliminated with the help of the DoubletFinder package (version 2.0.4). After the filtering process, a concluding set of 85 007 high‐quality cells was preserved for subsequent analysis.

Gene expression values were standardised utilising the LogNormalise approach featured in the NormaliseData function of Seurat, which adjusts each cell's total expression to 10 000 before applying a logarithmic transformation. The FindVariableFeatures function was utilised to identify the 2000 genes with the highest variability (highly variable genes [HVGs]). Following this, principal component analysis (PCA) was performed on the scaled data derived from these HVGs. A graph‐based clustering method, implemented through the FindClusters function, was employed to categorise cells based on the leading 20 principal components. For the purpose of two‐dimensional representation, Uniform Manifold Approximation and Projection (UMAP) was employed, utilising the same set of principal components.

The identification of marker genes within each cluster was conducted through the application of the FindAllMarkers function. The FindMarkers function was utilised to pinpoint differentially expressed genes (DEGs) under designated conditions, employing a criteria of adjusted *p*‐value < 0 .05 (Bonferroni correction) and an absolute log2‐fold change exceeding   0.58. To explore the functional significance of the DEGs, analyses for Gene Ontology (GO) terms and Kyoto Encyclopedia of Genes and Genomes (KEGG) pathways were performed, using the clusterProfiler R package that is based on the hypergeometric distribution.

Gene set enrichment analysis at the single‐cell level utilised Gene Set Variation Analysis (GSVA). Annotations for pathways were acquired from the official websites of the KEGG and GO databases (https://www.kegg.jp/ and https://geneontology.org/) and formatted to ensure compatibility through the GSEABase R package (version 1.44.0). Subsequently, the GSVA method was applied to the normalised expression data of individual single cells, employing the GSVA R package (version 1.30.0) to produce an enrichment score for each pathway in every single cell.^21^ These scores represent the relative activity level of each biological pathway within individual cells. Differences in pathway activity between predefined cell groups or experimental conditions were subsequently assessed using the LIMMA R package (version 3.38.3).

Intercellular communication networks were inferred by the CellChat R package (version 2.1.2).^22^ The normalised gene expression matrix and cell metadata (containing cell‐type annotations) were used as input to create a CellChat object. The standard workflow was executed as follows: the identify Overexpressed genes and identify Overexpressed interactions functions were used to identify potential ligand‒receptor interactions, followed by data projection onto a protein‒protein interaction network. Communication probabilities were then calculated (compute CommunProb), filtered for interactions involving cell groups with fewer than 10 cells (filter Communication), and aggregated at the pathway level (compute CommunProb Pathway). Finally, the inferred cell‒cell communication network was summarised using the aggregate Net function.

#### 2.2.2 Spatial transcriptomics sequencing analysis

Mouse lung specimens harvested freshly were dissected, and any moisture on the surface was eliminated using cleanroom wipes. Optimal cutting temperature (OCT) compound (SAKURA, catalogue no. 4583) was used to embed the tissues, which were then quickly frozen using dry ice and stored at ‒80°C until further processing. All library preparation and sequencing tasks that followed were carried out by Shanghai OE Biotech Co., Ltd. The workflow adhered to the established protocol of the 10× Genomics Visium Spatial Gene Expression system. In summary, OCT‐embedded tissue samples were cryosectioned into 10‐µm thick slices utilising a Leica CM1950 cryostat (Leica Microsystems) set at ‒20°C. This procedure comprised two major phases: (1) tissue optimisation—the sections were placed on Visium Spatial Tissue Optimisation Slides (10× Genomics, catalogue no. PN‐1000193) to ascertain the optimal time for tissue permeabilisation, in accordance with the manufacturer's guidelines (CG000238). (2) Library preparation—for the conclusive experiment, serial sections were mounted onto Visium Spatial Gene Expression Slides (10× Genomics, catalogue no. PN‐1000184/1000185). The slides underwent methanol fixation, haematoxylin and eosin (H&E) staining, followed by bright‐field imaging. Subsequent to imaging, tissue permeabilisation, cDNA synthesis, amplification and library construction were performed using Visium Spatial Gene Expression reagents and protocols (CG000239 and CG000160). The resulting libraries for sequencing were processed on a BGI DNBSEQ‐T7 platform utilising a paired‐end 100 bp (PE100) configuration.

Processing of the raw sequencing data in FASTQ format was conducted using version 2.0.1 of the Space Ranger software pipeline from 10× Genomics. The alignment of the reads was executed against the mouse reference genome mm39 (GRCm39), with UMI counts compiled for each spatially barcoded location. The identification of spots corresponding to the tissue sections was carried out automatically by utilising the associated histology images for subsequent analysis.

The Seurat R package (version 4.1.0) was subsequently used to import the filtered feature‒barcode matrix for further analysis.^22^ The SCTransform function was utilised simultaneously for data normalisation, variance stabilisation and identifying the top 3000 HVGs.^23^ A dimensionality reduction was performed using PCA on the scaled data pertaining to these HVGs.

To account for potential technical variations between samples, the RunHarmony function from the Harmony R package (version 1.0) was applied to integrate the datasets and remove batch effects.^24^ The harmonised principal components were subsequently employed for graph‐based clustering via the Find Clusters function and for non‐linear dimensionality reduction using UMAP with the Run UMAP function.

The FindAllMarkers function, using the ‘bimod’ test, was employed to identify marker genes for every cluster. The FindMarkers function, employing the ‘presto’ test, was then used to assess differential expression under specific conditions. Genes exhibiting an adjusted *p*‐value of less than 0 .05 (post‐Bonferroni correction) and an absolute log2‐fold change exceeding  0.58 were deemed statistically significant. Following this, enrichment analyses for GO and KEGG pathways were conducted on DEGs, using a hypergeometric test performed in R (version 4.0.3). All computational analyses were executed by OE Biotech Co., Ltd.

To clarify the composition of cell types present within each spot of the ST analysis, we utilised Robust Cell Type Decomposition (version 1.1.0).^25^ The method was applied using the paired scRNA‐seq dataset from the same mouse lung model as the reference. The analysis was run with the doublet_mode parameter set to FALSE. All other parameters were kept at their default settings, except for the minimum number of cells per cell type, which was set to >1, and the minimum UMI count per spot (pixel), which was set to >1. The computational deconvolution was performed by Shanghai OE Biotech Co., Ltd.

### 2.3 In vivo lung monitoring in mice

#### 2.3.1 Mouse lung function testing

Pulmonary function was evaluated in all experimental mouse cohorts (N: control group, S: silicosis group, A: silicosis + AMD3100, and AMD3100: AMD3100‐treated group) via whole‐body plethysmography. Each mouse was positioned in the plethysmograph chamber and given a 30‐min acclimation period. Subsequently, respiratory parameters were continuously monitored for 60 min while the animals remained conscious and unrestrained. The main metrics evaluated included tidal volume (*V*_t_), minute ventilation (*M*_v_) and the expiratory flow measured at 50% of the tidal volume (EF50). Statistical analyses comparing these measures across groups were conducted to determine any significant variations.

#### 2.3.2 Micro‐computed tomography

High‐resolution micro‐computed tomography (CT) imaging was carried out with a Hiscan XM system (Suzhou Hiscan Information Technology Co., Ltd.). During in vivo scanning, mice were anaesthetised by inhaling 1.5%‒2% isoflurane in 100% oxygen and placed on their backs in the imaging chamber. Ex vivo scans were also performed on fixed lung tissue specimens positioned directly within the scanner.

The scanner was calibrated prior to use by imaging a phantom containing standard references of air (1.5 mL) and water (50 mL), setting the Hounsfield unit (HU) scale to ‒1000 and 0 HU, respectively. Scans were obtained using the subsequent parameters: an X‐ray tube voltage of 80 kV, a current of 100 µA, an exposure duration of 50 ms for each step, and a rotation step of   0.5° across a complete 360° rotation.

For analysis, the central slice of each scan was selected, and the average HU within the volume of interest was measured. The mean HU value was used as a quantitative indicator to assess the progression of pulmonary inflammation and fibrosis.

### 2.4 Immunofluorescence staining

Lung tissue samples that were preserved through the method of fresh freezing underwent slicing into 8‐µm sections, a process conducted with the assistance of a cryostat. Once the sections were prepared, they were subjected to a blocking procedure lasting 2 h at room temperature. This blocking was performed using a solution specifically formulated with 10% goat serum and 0.3% Triton X‐100 to minimise nonspecific binding of antibodies. Following the blocking step, the sections were incubated overnight at a temperature of 4°C with a selection of primary antibodies. The antibodies used in this phase included rabbit anti‐Cxcl12 (catalogue no. 17402‐1‐AP from Proteintech), rabbit anti‐α‐smooth muscle actin (α‐SMA, catalogue no. ab124964 from Abcam) and mouse anti‐Cxcr4 (catalogue no. ab1670 also from Abcam). This overnight incubation was crucial for allowing the primary antibodies to effectively bind to their respective target antigens within the lung tissue sections. After thoroughly washing the sections to remove any unbound primary antibodies, the samples were treated with suitable fluorescent secondary antibodies. This incubation occurred for a duration of 2 h at room temperature, enabling visualisation of the bound primary antibodies through fluorescence. Subsequently, 4',6‐diamidino‐2‐phenylindole (DAPI) was used for nuclear staining, providing a contrast that facilitated the identification of cell nuclei in the images. Finally, the resultant images were captured using an FV3000 confocal laser scanning microscope produced by Olympus, ensuring high‐resolution imaging of the processed lung tissue sections.

### 2.5 Flow cytometry

Blood was drawn from the orbital sinus of the mouse. To lyse red blood cells, the whole blood was combined with RBC lysis buffer in a 1:3 (v/v) ratio and incubated for 10 min at 4°C. After centrifugation, the supernatant was removed. This lysis procedure was repeated until all red blood cells were eliminated. The remaining cells were then washed and resuspended in a solution of 3% bovine serum albumin (BSA) in PBS. For staining cell surfaces, the cell suspension was treated with fluorochrome‐labelled primary antibodies, diluted as per the manufacturer's protocol, and incubated for 1 h at 4°C in darkness. Post‐incubation, the cells underwent two washes with 3% BSA to eliminate any unbound antibodies before being resuspended in a suitable volume of the same buffer for subsequent analysis. Data collection was carried out promptly using a FACS Canto II flow cytometer from BD Biosciences, operated with Cell Quest Pro software. The resulting data were then analysed using FlowJo software (version 7.6.5 from Tree Star).

### 2.6 Histopathological staining

The lobes from the left lung were collected and preserved in 4% paraformaldehyde, then embedded in paraffin and sliced into 4 µm thick sections. Subsequently, these sections underwent incubation at 60°C for 3 h, followed by deparaffinisation with xylene, rehydration through a gradient of alcohol solutions, and washing with ddH_2_O. Pathological assessments and evaluations of fibrosis were performed using H&E staining, in addition to Masson's trichrome staining.

### 2.7 Cell grouping and pretreatment and transwell migration assay

Cell migration was evaluated through a transwell assay employing inserts with 8 µm pores. MH‐S and RAW 264.7 cells underwent a 24 h pretreatment with or without a 200 µg/mL silica suspension to create silica‐treated experimental groups and untreated control groups. For the transwell assay, our goal was to observe the migration of RAW or MH‐S cells within 24 h; at 200 µg/mL, the damage to cell proliferation is relatively minor, while the degree of migration induced is more pronounced. Post‐pretreatment, the cells were collected and resuspended in serum‐free medium. Cells were counted using a haemocytometer with Trypan Blue exclusion (viability > 95%) prior to seeding, ensuring that exactly 2 × 10^5^ viable cells were loaded into each upper chamber. This consistency in seeding ensures that the absolute counts of migrated cells per field are directly comparable across experimental groups. The lower chamber received 600 µL of complete medium supplemented with 100 ng/mL Cxcl12 (PeproTech), as recommended on the official website to serve as a chemoattractant (https://www.medchemexpress.cn/recombinant‐proteins/cxcl12‐sdf‐1‐beta‐protein‐mouse.html). Complete medium lacking Cxcl12 was utilised for the negative control setup. The plates were later incubated at 37°C for 24 h in an atmosphere containing 5% CO_2_. After the incubation period ended, any non‐migratory cells remaining on the upper side of the membrane were gently eliminated using a cotton swab. Cells that had migrated to the lower surface of the membrane were then fixed with 4% paraformaldehyde for 30 min and later stained with a 0 .1% crystal violet solution for 10 min. Following a rinse with PBS, five randomly chosen fields from each membrane were captured at a magnification of 200× using an inverted light microscope (Nikon Eclipse Ti). Data are expressed as the mean number of migrated cells per high‐power field.

### 2.8 Cell seeding and transfection

Twenty‐four hours prior to transfection, seed approximately 3 × 10^5^ L929 cells per well in a six‐well plate using complete culture medium containing 100 ng/mL of recombinant TGF‐β protein (MCE, HY‐P70648). The following day, the cell density should reach 70%–80%.

Prior to transfection, replace the medium in each well with 2 mL of fresh complete culture medium. Add 125 µL of dulbecco's modified eagle medium (DMEM) culture medium without antibiotics or serum and 100 pmol of siRNA‐181 to each well of cells and gently mix using a pipette. Then, add 4 µL of the LipoRNAi transfection reagent (Biyun Tian, C0535) and gently mix again. Incubate at room temperature for 20 min and then continue culturing for 48 h. Then, 5 × 10^4^ MH‐S cells were seeded into the upper chamber of the transwell insert according to the specifications of each experimental group. For the silica group (Sil), cells were pretreated for 24 h with medium containing 200 µg/mL silica dioxide prior to seeding.

Following the migration assay, the chambers were carefully taken out, rinsed with PBS, and subjected to fixation using 4% paraformaldehyde for a duration of 30 min. Afterwards, the chambers were washed and treated with crystal violet in the dark for another 30 min. Following staining, cells on the inner surface of the chamber were gently removed using a cotton swab. The chambers were then placed on microscope slides, and five random fields per chamber were photographed at 200× magnification for cell counting. Meanwhile, transfected cells were collected and the RNA was extracted using the chloroform method with TRIzol (Thermo Fisher Scientific, 15596018CN). The RNA level knockdown efficiency was detected via qPCR (Table 1).

**TABLE 1:** siRNA‐181 primer sequences (from GeneUniversal).

PIN	Sequence (5′ to 3′)
A1041802	CGGUAAACCAGUCAGCCUGTT
A1041803	CAGGCUGACUGGUUUACCGTT

### 2.9 Statistical analysis

The software GraphPad Prism (version 8.0.2) was used to perform the statistical analysis. To evaluate the differences between two groups, a Student's *t*‐test was applied. In instances where comparisons involved three or more groups, either one‐way or two‐way analysis of variance was utilised. The findings are expressed as the mean ± standard error of the mean. A *p*‐value less than  .05 was deemed statistically significant.

## 3 RESULTS

### 3.1 The blockade of Cxcr4 by AMD3100 significantly ameliorates silicosis in mice

**FIGURE 1:**
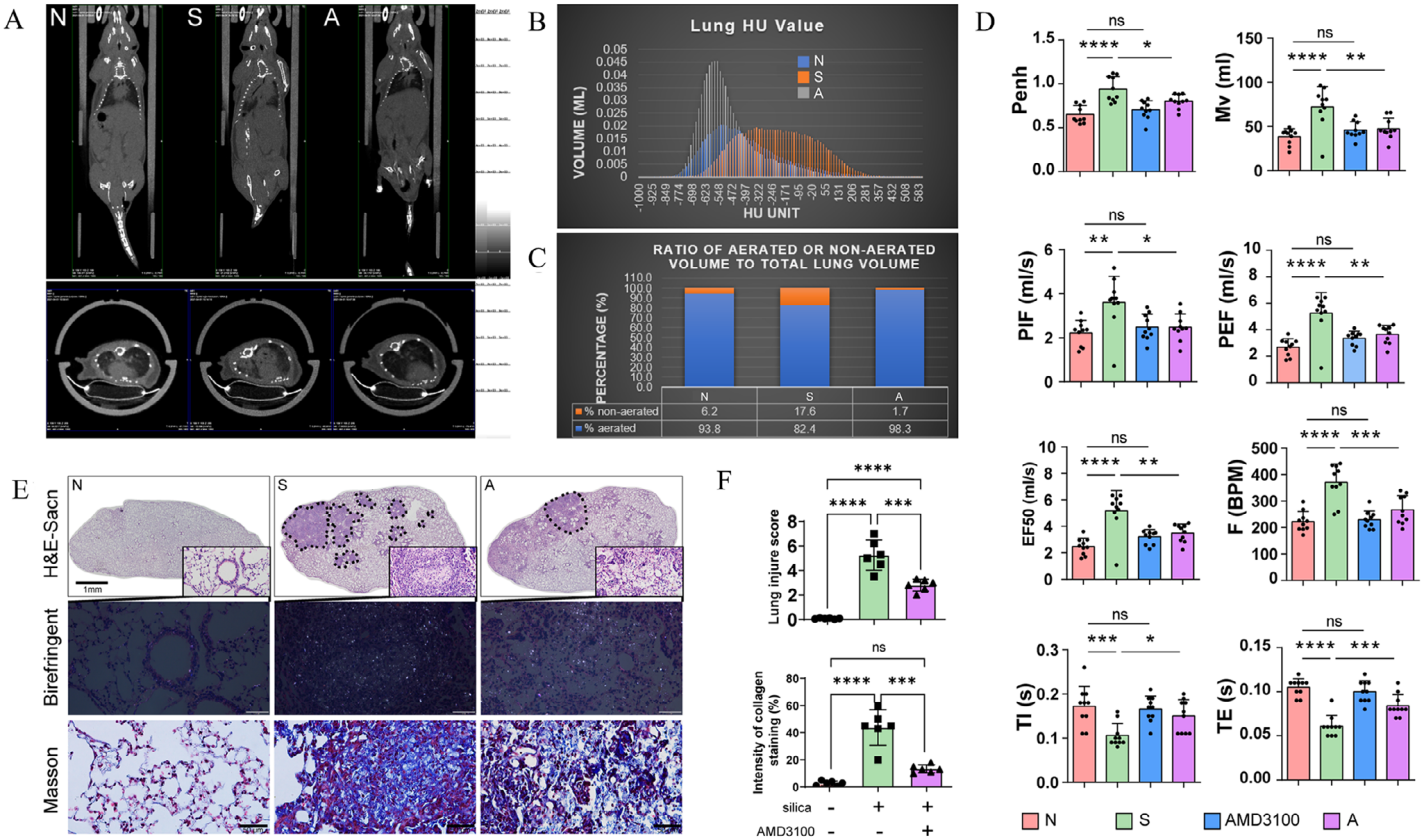
Therapeutic role of the Cxcr4 inhibitor AMD3100 in a mouse model of silicosis. (a‒c) Computed tomography (CT) scans and corresponding quantitative analyses illustrate the effect of AMD3100 on lung damage induced by silica exposure (scale bar = 1 mm). (d) Bar chart display of quantitative indicators for pulmonary function assessment. (e) Histological examination of lung tissues reveals patterns of silica accumulation and inflammation, visualised using haematoxylin and eosin (H&E) staining (scale bar = 1 mm), birefringent staining (scale bar = 50 µm) and Masson's trichrome staining (scale bar = 50 µm). (f) Quantify the extent of lung injury using the lung injury score (LIS). (g) Comparative analysis of collagen staining intensity ratios in lung tissues across different experimental groups. Statistical analyses were performed by one‐way analysis of variance (ANOVA). ^*^ *p* < .05, ^**^ *p* < .01.

To assess the therapeutic efficacy of the Cxcr4 antagonist AMD3100 in the context of silicosis, we performed CT scans on murine lung tissues. The findings demonstrated that following exposure to SiO_2_, there was a manifestation of high‐density opacities and substantial silica particle accumulation within the pulmonary structures of the mice, indicative of pronounced lung tissue damage (Figure 1a), along with a marked decrease in lung ventilation (Figure 1b,c). The monitoring of lung function in mice exposed to SiO_2_ revealed a significant increase in enhanced pause, minute ventilation (mL), maximum inspiratory flow rate (mL/s), maximum expiratory flow rate (mL/s) and respiratory rate (breaths/min). These findings suggest that airway narrowing, likely due to inflammation induced by SiO_2_ exposure, reduced the effective ventilation area, resulting in various physiological changes. Additionally, the marked increase in inspiratory time (s), expiratory time (s) and cumulative volume (mL) indicates compromised lung function, with lung consolidation reducing the available space for gas exchange. Consequently, the mice extended their breathing time to maintain adequate oxygen supply to the body (Figure 1d). Histopathological staining results corroborated these observations (Figure 1e). Compared to the control group (N), the extent and severity of lung damage in SiO_2_‐exposed mice were significantly greater; however, treatment with AMD3100 markedly ameliorated lung damage in these mice (Figure 1f). In the initial phases of acute lung injury, the overactivation of lung fibroblasts results in fibrotic proliferation and collagen synthesis, thereby initiating lung fibrosis.

Analysis of collagen in mice demonstrated that lung tissue fibrosis was considerably more severe in those exposed to SiO_2_ compared to the N group. In contrast, the A group showed a substantially reduced level of fibrosis relative to the S group (Figure 1g). These results suggest that the Cxcr4 antagonist AMD3100 has a significant therapeutic potential against silicosis. We propose that increased Cxcr4 expression is a key factor in the development of silicosis.

### 3.2 Single‐cell transcriptomics uncovers how AMD3100 influences the cellular environment in a mouse model of silicosis

**FIGURE 2:**
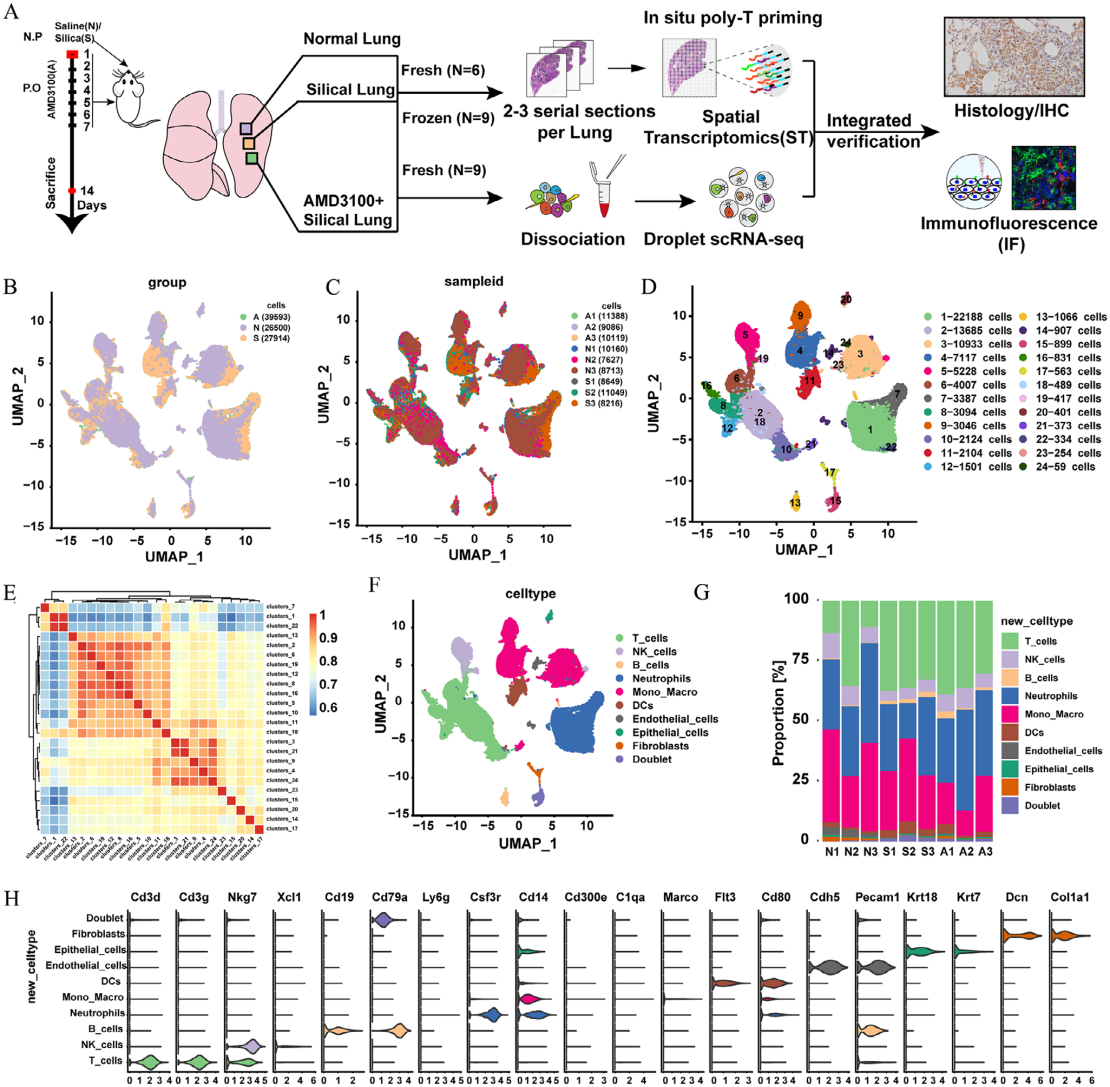
Single‐cell RNA sequencing reveals 24 distinct cellular populations within the lungs of mice afflicted with silicosis. (a) Schematic diagram of establishing an AMD3100‐treated silicosis mouse model and subsequent multi‐omics sequencing processing. (b‒d) The t‐distributed Stochastic Neighbor Embedding (t‐SNE) plot displays the major cell types and cell count summary obtained from unsupervised clustering analysis of single cells from silicosis mouse lung tissue, along with the distribution of each cell type. (e) Clustering analysis based on correlation of the 24 identified cell clusters. (f) Distribution of the identified cell types on the t‐SNE plot. (g) Proportional representation of cells across samples. (h) The violin plot illustrates the expression patterns of cell‐type‐specific markers (*n* = 3 per group).

To explore the diverse cell types present in mouse lung tissue and to analyse the variations and relationships between different cell populations, we performed scRNA‐seq on nine lung tissue samples from mice. The samples were divided into three categories: N, S and A, with three samples in each group, leading to the development of a detailed scRNA‐seq atlas (Figure 2a). After applying initial quality control procedures, we obtained scRNA‐seq data from 26 500 cells in the N group, 27 914 cells in the S group and 39 593 cells in the A group (Figure 2b,c). To assess the cellular makeup related to silicosis, we conducted PCA on the variable expression genes from all cells, revealing 24 primary cell clusters. These clusters were then further investigated using correlation‐based clustering analysis (Figure 2d,e). Subsequently, differential gene expression analysis was conducted to identify cell‐cluster‐specific marker genes, enabling the characterisation of each cell cluster. This analysis identified a total of 10 distinct types of lung tissue cells (Figure 2f). The following cell populations were clearly identified: fibroblasts (Dcn^+^, Col1a1^+^), epithelial cells (Krt18^+^, Krt17^+^), endothelial cells (Cdh5^+^, Pecam1^+^), dendritic cells (Cd80^+^, Flt3^+^), mono‐macrophages (Cd14^+^), neutrophils (Cst3r^+^, Cd14^+^), B cells (Cd79a^+^, Pecam1^+^), natural killer cells (Nkg7^+^), T cells (Cd3^+^, Cd3g^+^) and doublet populations (Cd79a^+^) (Figure 2h). Among these, T cells, monocytes and fibroblasts exhibited a significant increase in numbers following silica exposure, whereas neutrophils showed a decrease. Administration of AMD3100 led to a decrease in the quantity of mononuclear macrophages and neutrophils within the lung tissue of mice. This suggests that extended silica exposure triggers persistent inflammatory reactions in the lungs, which in turn facilitates the initiation and advancement of pulmonary fibrosis. By reducing the buildup of mononuclear macrophages and easing pulmonary inflammation, AMD3100 could potentially lessen or suppress the development of pulmonary fibrosis (Figure 2g,h). To summarise, we discovered multiple cell populations exhibiting unique distribution profiles across various tissue specimens. These populations are likely to have a significant function in the mouse model of silicosis.

### 3.3 In the context of pulmonary fibrosis, macrophages and fibroblasts demonstrate a close spatial correlation

**FIGURE 3:**
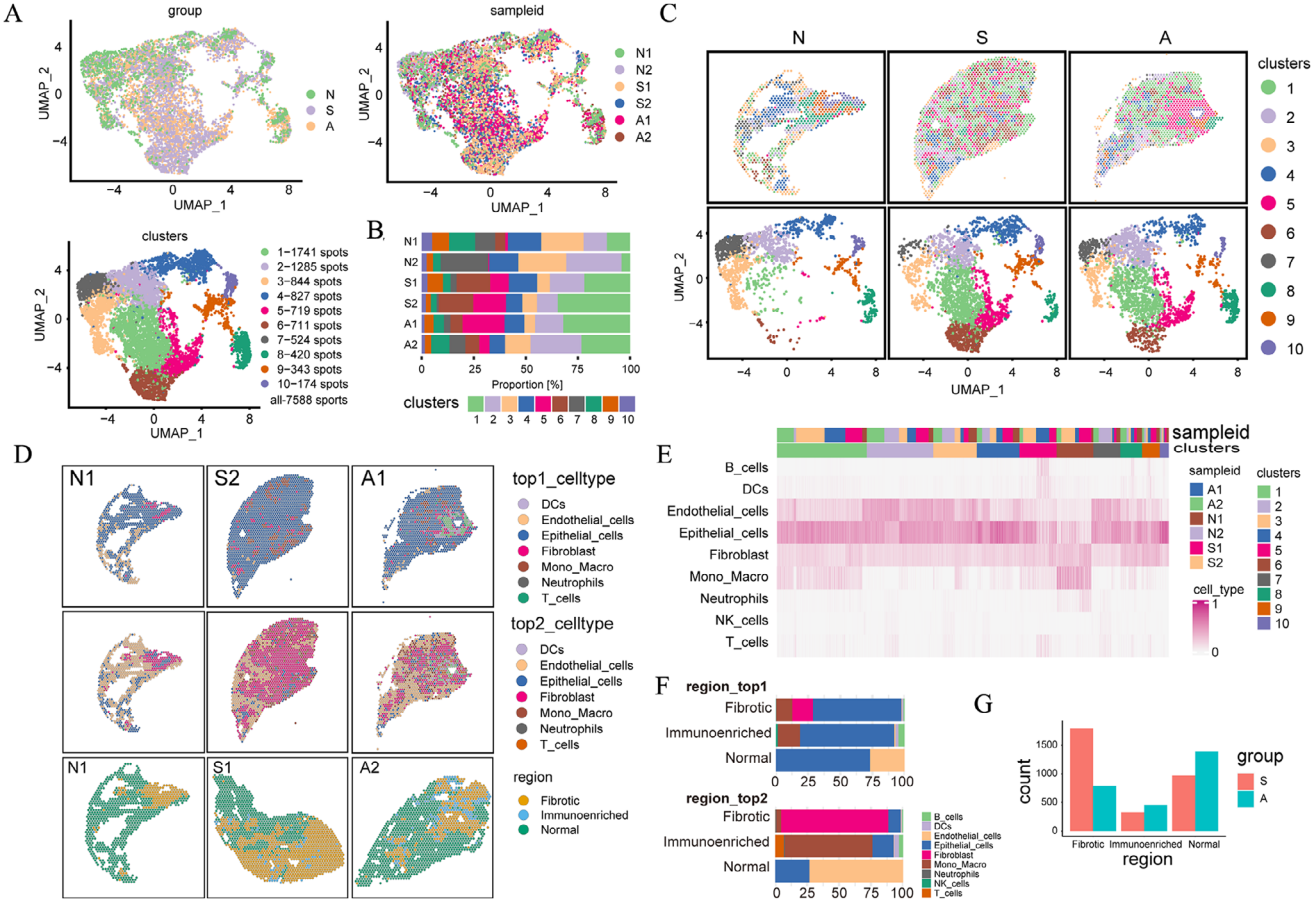
Characterisation of cellular samples using spatial transcriptomics. (a) Unsupervised clustering of individual spots from mouse models of silicosis, with group, sample and cluster information visualised in a t‐distributed Stochastic Neighbor Embedding (t‐SNE) plot. (b) Mapping of representative tissue sample capture areas to illustrate Spot cluster distribution in a t‐SNE plot for each group. (c) Distribution of Spot clusters across each sample. (d) Spatial distribution of the two most prevalent cell types within the tissue capture area. (e) Heatmap illustrating Spot cluster mapping based on the copy number of cellular markers obtained through deconvolution. (f) Classification of tissue into normal, inflammatory and fibrotic spots according to the deconvolution algorithm. (g) Technical comparison of spots in normal, inflammatory and fibrotic regions (*n* = 2 per group).

To thoroughly investigate the spatial distribution characteristics of cells in silicosis, we obtained lung tissue specimens from six mice, comprising two normal control groups (N1 and N2), two groups exposed to SiO_2_ (S1 and S2), and two groups subjected to both silica exposure and AMD3100 treatment (A1 and A2). The lesion areas of each sample were scanned and analysed using H&E staining alongside gene expression profiling, resulting in the identification of 10 distinct spots (Spot). The clustering proportion of each Spot varied among the samples (Figure 3a‒c). By dividing lung tissue into distinct areas according to gene expression patterns and recognising that each sampling spot contains multiple cells, we utilised a feature‐based method to combine ST with scRNA‐seq data to approximate the different cell types present at every capture location. The results showed a considerable rise in macrophage numbers within the fibrotic areas of the lungs in silica‐exposed mice relative to the N group. After administration of AMD3100, a decrease in the macrophage proportion was noted, suggesting that macrophages could have a central function in the development of pulmonary fibrosis (Figure 3d). Importantly, macrophages and fibroblasts exhibit a high level of spatial co‐localisation and gather notably in zones of SiO_2_ accumulation in lung tissue. Additionally, it is clear that the count of epithelial cells in the non‐affected lung regions of silica‐exposed mice is substantially reduced, while the number of neutrophils is greatly increased, implying their role in driving inflammation (Figure 3f,g). Therefore, we suggest that the rise in macrophages might be a key element in triggering pulmonary fibrosis.

### 3.4 Identifying different macrophage populations in silicosis

**FIGURE 4:**
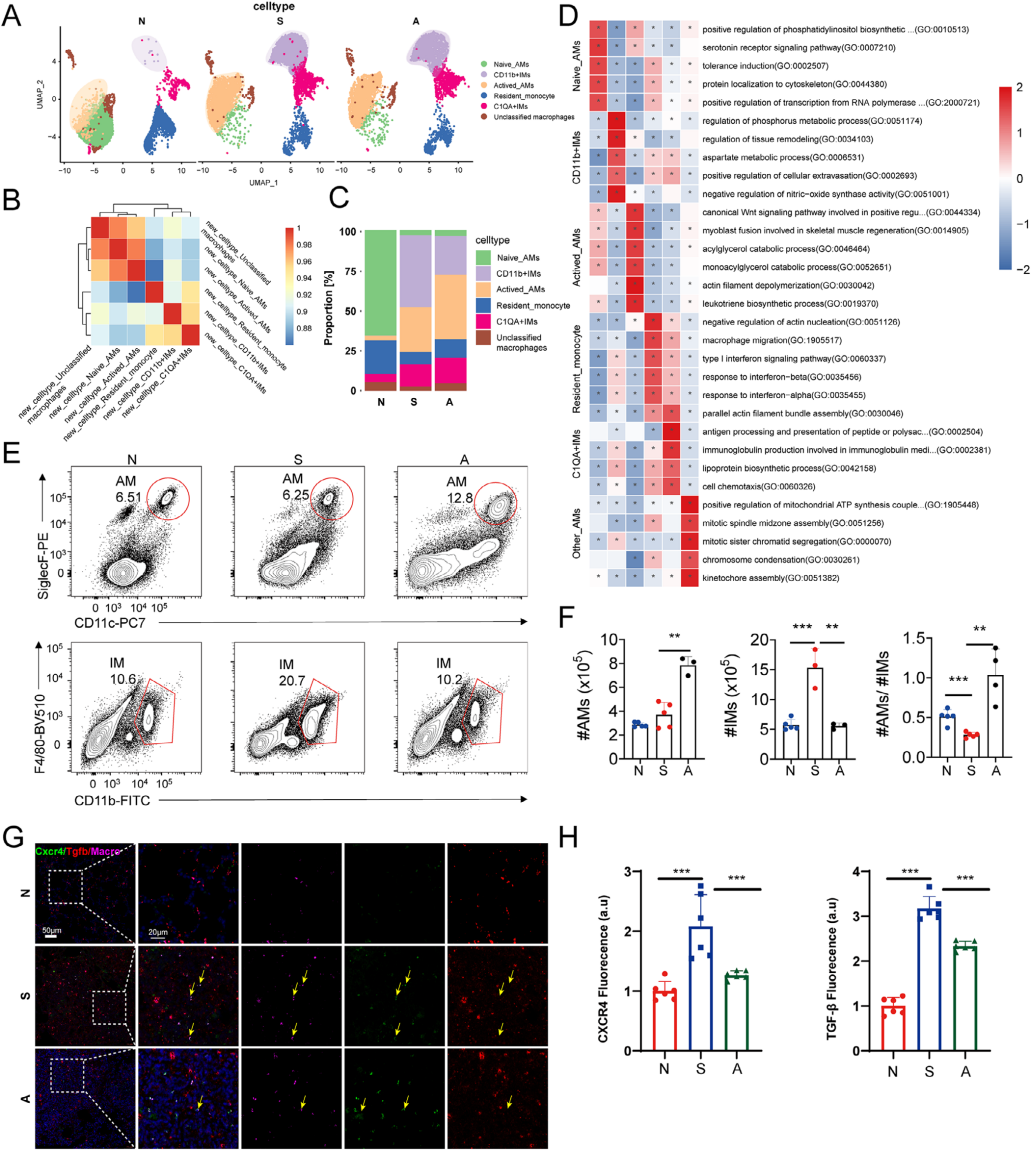
Macrophage subpopulations and classification in silicosis. (a) The t‐distributed Stochastic Neighbor Embedding (t‐SNE) visualisation illustrating distinct macrophage subpopulations across experimental groups. (b and c) The quantitative assessment of the proportional representation of each subpopulation within each group, and the heatmap showcasing characteristic marker genes for these subpopulations. (d) Gene Ontology (GO) pathway enrichment analysis. (e and f) Flow cytometry was employed to validate gating strategies for isolating subpopulations and measuring the relative proportions of alveolar macrophages (AMs) and interstitial macrophages. (g and h) Representative immunofluorescence staining images depicting the expression of Tgfb1 in Cxcr4^+^ AMs. Statistical analyses were performed by one‐way analysis of variance (ANOVA). ^*^ *p* < .05, ^**^ *p* < .01; N group: *n* = 5, S group: *n* = 5, A group: *n* = 4.

To explore the connection between macrophages and fibroblasts and to further validate the essential function of macrophages in silicosis, we conducted an examination of macrophage subpopulations. Utilising scRNA‐seq data, we identified and categorised macrophage subpopulations into six distinct groups: naive macrophages (naive‐AMs), Cd11b^+^ interstitial macrophages (Cd11b^+^ IMs), activated macrophages (Activated_AMs), resident monocytes (Resident_Monocyte), C1qa^+^ macrophages (C1qa^+^ IMs) and other macrophages (unclassified macrophages) (Figure 4a). Following exposure to SiO_2_, there was a marked increase in the number of macrophages in the lungs of mice, including the proliferation of IMs and AMs, which further facilitated the onset and progression of pulmonary fibrosis. Notably, the number of IMs was particularly elevated. Treatment with AMD3100 significantly reduced the accumulation of IMs, thereby mitigating the progression of pulmonary fibrosis to some extent (Figure 4b,c). Further analysis indicated a substantial increase in the number of activated AMs in mouse lung tissue, with associated GO pathways primarily related to the regulation of fibroblast aggregation (GO:0014905) and neutrophil synthesis (GO:0019370) (Figure 4d). Flow cytometry results demonstrated a significant increase in both AMs and IMs following SiO_2_ exposure. Initially, live single leukocytes (Cd45^+^) were gated, followed by the selection of Cd11b^+^Ly6g^−^ myeloid cells. AMs were characterised as Ly6c^−^Cd11b^−^ Siglec F^+^Cd11c^+^ cells and IMs as F4/80^+^Cd11b^+^ cells. Neutrophils, identified as Cd11b^+^Ly6g^+^, served as a reference population (Figure S1). Additionally, the proportion of neutrophils increased partially after SiO_2_ exposure and decreased following AMD3100 treatment. Following exposure to SiO_2_, the quantity of AMs did not exhibit a statistically significant increase; however, a significant increase was observed subsequent to AMD3100 treatment. Conversely, the number of IMs significantly rose after SiO_2_ exposure and significantly declined following AMD3100 treatment. Consequently, the AMs/IMs ratio experienced a significant reduction after SiO_2_ exposure and a significant elevation after AMD3100 treatment. These findings imply that IMs are more stringently regulated post‐SiO_2_ exposure, whereas AMs undergo only moderate regulation in terms of their numbers (Figure 4e,f). Of particular interest is the Cxcr4^+^ AM population, which demonstrates relatively high expression levels of TGF‐β in group S, yet these levels diminish in group A (Figure 4g,h).

### 3.5 AMD3100 treatment downregulates expression of Cxcl12 in activated fibroblasts

**FIGURE 5:**
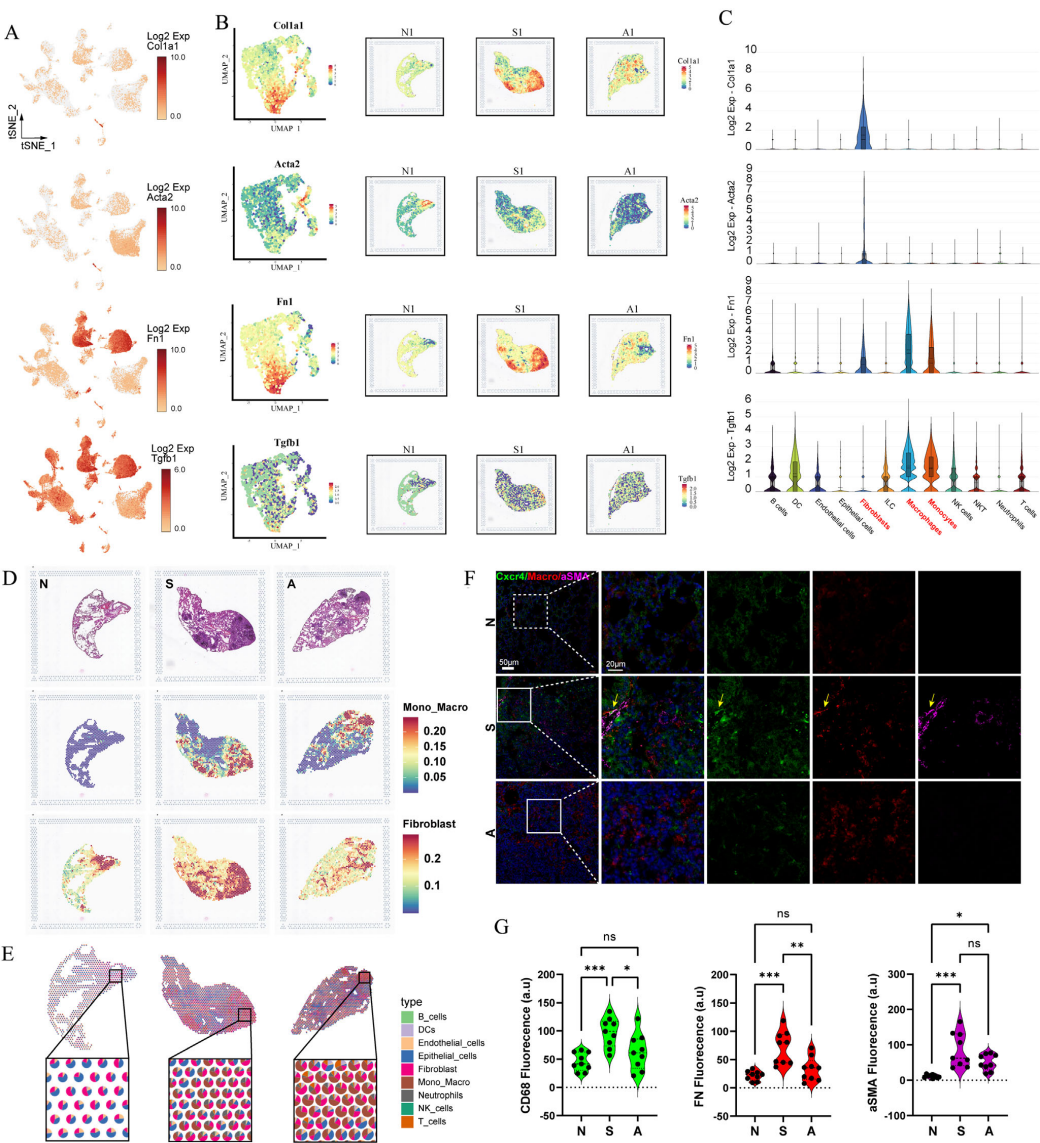
AMD3100 mitigates pulmonary fibrosis by suppressing fibroblast activation and macrophage recruitment. (a) A feature plot illustrating the expression of characteristic genes (Col1a1, Acta2, Fn1, Tgfb1) derived from single‐cell sequencing data. (b) Spatial expression profiles of these characteristic genes across different experimental groups. (c) The violin plot illustrating the expression profiles of signature genes among diverse cell populations. (d) Visualisation of spatial transcriptomics highlighting the expression patterns of fibroblasts and macrophages across different groups (*n* = 2 per group). (e) The proportion of distinct cell types expressed per spot in the spatial transcriptome. (f) Representative images from immunofluorescence staining illustrating the spatial interaction between fibroblasts and macrophages. (g) Correlation analysis accompanied by quantitative findings. Statistical analyses were performed by one‐way analysis of variance (ANOVA). ^***^ *p* < .001, ^**^ *p* < .01, ^*^ *p* < .05; *n* = 3 per group.

Upon exposure to SiO_2_, fibrotic lesions demonstrated a notable accumulation of fibroblasts, aligning with the recruitment and activation of myofibroblasts aimed at encapsulating the silica particles. This phenomenon was associated with a pronounced upregulation of TGF‐β within the fibrotic regions, indicative of a strong inflammatory and pro‐fibrotic response. Administration of AMD3100 significantly reduced this inflammation and cellular recruitment, thereby alleviating the overall fibrotic pathology (Figure 5a‒c). ST analysis further identified a high degree of spatial co‐localisation between pro‐fibrotic macrophages and activated fibroblasts, a finding substantiated by immunofluorescence staining. Notably, treatment with AMD3100 significantly disrupted this spatial clustering of macrophages and fibroblasts (Figure 5d‒g). These findings collectively indicate that exposure to SiO_2_ activates macrophages, which subsequently phagocytose particles and secrete factors that recruit and activate fibroblasts, thereby initiating an encapsulation process. The administration of AMD3100 mitigates fibrosis by disrupting this essential macrophage aggregation and the subsequent interaction with fibroblasts.

### 3.6 Macrophages crosstalk with fibroblasts via Cxcl12/Cxcr4 signalling axis

**FIGURE 6:**
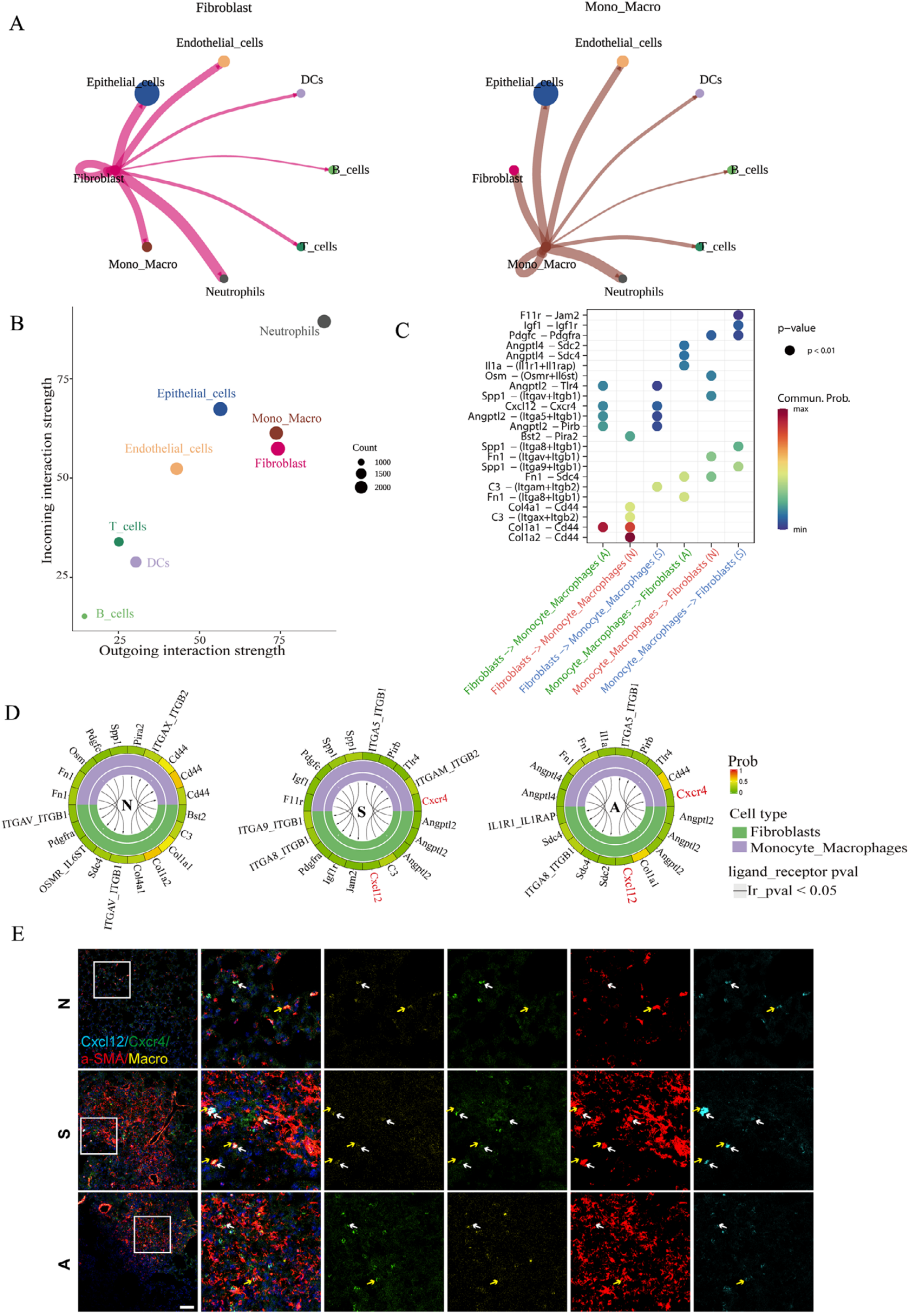
Intercellular communication between fibroblasts and macrophages. (a) Assess the frequency of communication between macrophages, fibroblasts and other cell types. (b) The primary functions of cells within the signalling network are analysed. The horizontal axis represents ligand communication strength, the vertical axis denotes receptor communication strength, and the size of the circles (count) indicates the number of ligand‒receptor pairs connecting this cell group to all other groups. (c and d) The bubble plot and the Circos plot are used to illustrate the pathways and their respective interaction strengths between Mo_Macrophage and fibroblast cells. (e) Representative immunofluorescence staining images reveal that α‐smooth muscle actin (α‐SMA)^+^Cxcl12^+^ fibroblasts (indicated by yellow arrows) recruit Macro^+^Cxcr4^+^ macrophages (indicated by white arrows). The scale bar represents 50 µm, with a sample size of *n* = 3 for each group.

Our analysis of cell‒cell interactions uncovered a robust communication network between macrophages and fibroblasts, indicating a pivotal role for macrophages in facilitating fibroblast activation and the development of pulmonary fibrosis (Figure 6a,b). Further investigation revealed a significant interaction specifically between monocyte‐derived macrophages (Mono_Macro) and fibroblasts, predominantly mediated by the Cxcl12/Cxcr4 chemokine axis in the silica‐exposed group (Figure 6c,d). This finding suggests that fibroblasts secrete Cxcl12 to recruit Cxcr4‐expressing Mono_Macro to fibrotic lesions. The effectiveness of this recruitment was confirmed through transwell assays, which demonstrated a pronounced migratory response of AMs to Cxcl12; this response was notably diminished in monocyte‐derived macrophages (Figure S3). These results highlight a primary role for AMs in driving fibrosis. Importantly, treatment with AMD3100, a Cxcr4 antagonist, effectively disrupted this cellular recruitment and mitigated fibrotic progression (Figure 6c‒e).

### 3.7 AMD3100 treatment inhibits Cxcl12^+^ fibroblast‐mediated chemotaxis of AMs and formation of silicotic nodules

**FIGURE 7:**
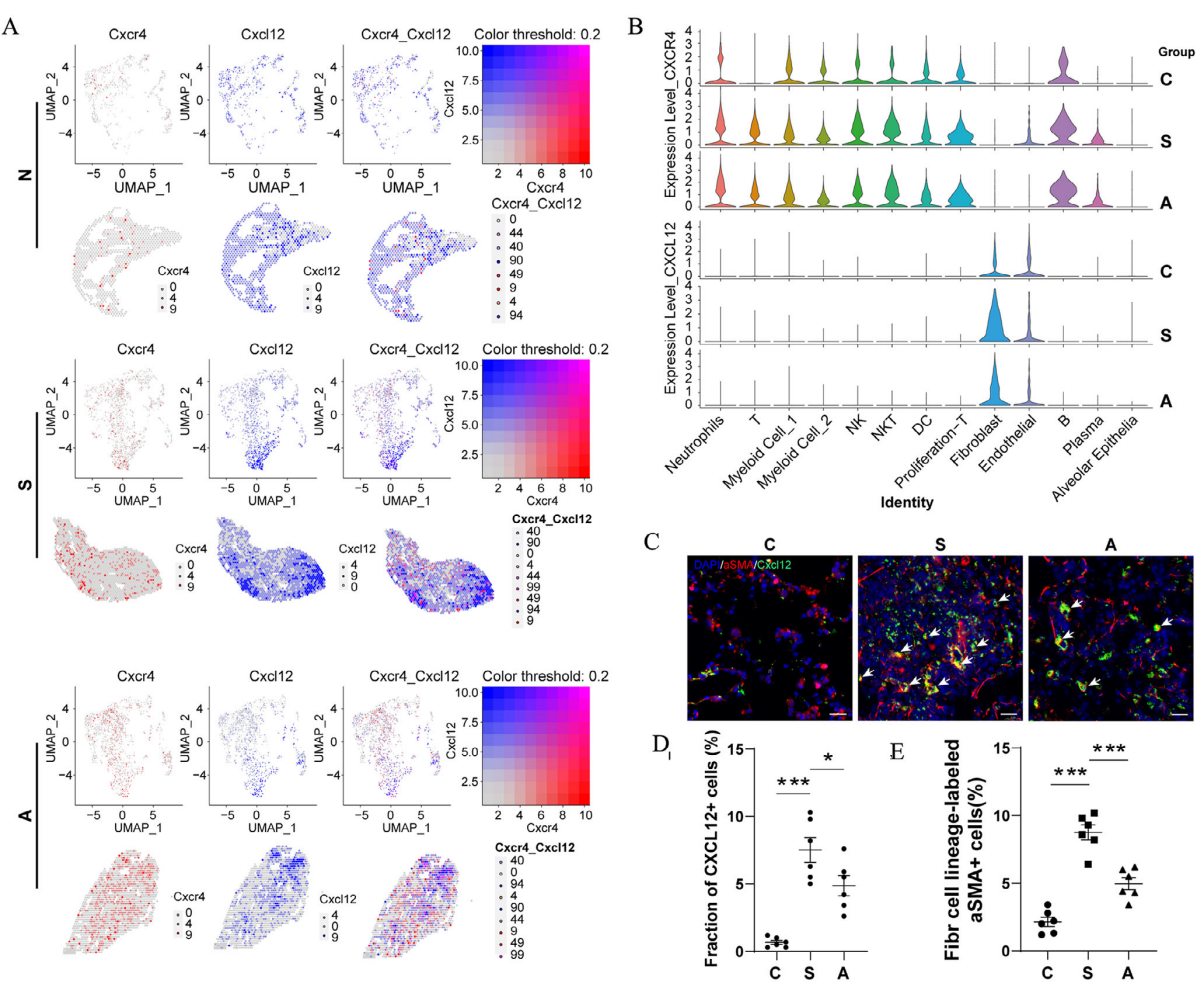
AMD3100 alleviates pulmonary fibrosis by inhibiting Cxcl12 expression in activated fibroblasts. (a) Spatial transcriptomics analysis revealed modifications in cell populations within murine lung tissue subsequent to SiO_2_ exposure and subsequent AMD3100 treatment. (b) The expression profiles of Cxcl12 and Cxcr4 were assessed across diverse cell types within the murine lung tissue. (c‒e) Immunofluorescence staining provided representative images of Cxcl12 expression in murine lung fibroblasts, accompanied by quantitative assessments. Statistical analyses were performed by one‐way analysis of variance (ANOVA). ^*^ *p* < .05, ^**^ *p* < .01, with *n* = 3 for each group.

Our research clarifies the essential function of the Cxcl12/Cxcr4 signalling pathway in the development of silica‐induced pulmonary fibrosis. Examination of fibrotic lung samples showed a marked increase in Cxcr4 levels within invading immune cells, including macrophages, neutrophils and T cells. In contrast, fibroblasts and endothelial cells demonstrated only negligible receptor expression. Notably, Cxcr4 expression was largely unaltered by AMD3100 treatment. In contrast, the expression of the ligand Cxcl12 was spatially restricted, primarily localised to stromal cells, including fibroblasts and endothelial cells (Figure 7a,b). Importantly, AMD3100 treatment significantly reduced Cxcl12 expression in fibroblasts, with a less pronounced effect in endothelial cells. This ligand‐specific downregulation was further supported by immunofluorescence analysis, which showed a marked induction of Cxcl12 in pulmonary fibroblasts following SiO_2_ exposure, an effect significantly attenuated by AMD3100 treatment (Figure 7c‒e). Collectively, our findings illuminate a central pathogenic mechanism whereby fibroblasts act as the primary source of Cxcl12, facilitating the recruitment of Cxcr4‐expressing immune cells to the site of injury. Our validation results utilising MH‐S cell lines in vitro corroborated the hypothesis that AMs exhibit elevated expression of TGF‐β and Cxcr4 following silica exposure. Subsequently, we treated the L929 cell line with TGF‐β. The intervention led to a significant increase in α‐SMA and Cxcl12 expression within fibroblasts (Figure S4). These observations indicate that AM activation occurs through the Cxcr4 signalling cascade, triggering TGF‐β release. This, in turn, promotes the transformation of fibroblasts into myofibroblasts, a process that advances the development of pulmonary fibrosis.

## 4 DISCUSSION

Silicosis is a progressive and ultimately fatal fibrotic lung disease for which no disease‐modifying therapies are currently available. Although dysregulated factors derived from macrophages are recognised as significant contributors, the precise cellular interactions that initiate and sustain fibrosis remain inadequately understood. Through an integrated multi‐omics approach that combines single cell and ST, our research suggest the presence of a self‐amplifying macrophage‒fibroblast circuit between macrophages and fibroblasts that drives the progression of silicosis. The principal findings of our study include the notable spatial co‐localisation of a specific pro‐fibrotic macrophage subpopulation with activated myofibroblasts within the fibrotic niche, as well as the identification of the Cxcl12/Cxcr4 chemokine axis as a central component of this pathogenic communication network.

The interplay between macrophages and fibroblasts is central to the control of fibrotic processes, and its presence across multiple organ systems underscores its essential nature. Research by Zhang et al. shows that within the setting of cardiac fibrosis linked to heart failure with preserved ejection fraction (HFpEF), Cxcr4^+^ macrophages serve as key drivers of inflammation and fibroblast stimulation, and their elimination reduces the advancement of the disease.^26^ From physical injury to radiation to autoimmunity, inflammation is the common pathway driving disease progression. This makes it a key therapeutic target: blocking inflammatory pathways could effectively treat a wide range of diseases.^27^, ^28^, ^29^ Correspondingly, recent studies indicate that within the tumour microenvironment of triple‐negative breast cancer, cancer‐associated fibroblasts exploit the Cxcl12/Cxcr4 signalling pathway to recruit monocytes and transform them into immune‐suppressive, lipid‐associated macrophages, thereby fostering a pro‐fibrotic niche.^30^, ^31^

ST validated scRNA‐seq cell types and visualised macrophage‒fibroblast interactions within lung microanatomy. Macrophages accumulated at silica deposits, while fibroblasts showed spatially consistent distribution, revealing their cooperative role in fibrosis. Previous studies have shown that macrophages play a pivotal role as orchestrators in the pathogenesis of pulmonary fibrosis, influencing disease progression through immune modulation, regulation of apoptosis and direct cellular interactions.^32^ In the context of silicosis, there is a notable shift in macrophage polarisation towards a persistent M2‐like phenotype, which collectively contribute to a pro‐fibrotic environment. Previous studies have clearly demonstrated that monocytes have been shown to significantly promote the progression of pulmonary fibrosis.^33^ However, a detailed characterisation of the Cxcr4^+^ macrophage subset remains lacking. Therefore, systematic identification and functional analysis of Cxcr4^+^ macrophage subpopulations using single‐cell sequencing technology would not only fill an important gap in our current understanding but also offer new insights into the immune mechanisms underlying pulmonary fibrosis. Such an approach may further provide key clues for the development of novel diagnostic markers and therapeutic targets.

Our study introduces a critical, spatially resolved perspective to this understanding. We identify a distinct subpopulation of Cxcr4^+^ macrophages that spatially co‐localises with fibroblasts within fibrotic niches, acting as a key driver of the disease. This finding is consistent with previously characterised pro‐fibrotic macrophage populations, such as the Mmp12hi subset in silicosis and monocyte‐derived macrophages that repopulate the lung in bleomycin‐induced fibrosis models.^7^, ^34^ The involvement of Cxcr4^+^ macrophages in promoting pathological fibrosis is consistently observed across various conditions, such as cancer and myocardial disease.^26^, ^30^ This study elucidates a self‐perpetuating cycle mediated by this axis: silica‐activated AMs secrete TGF‐β, which stimulates fibroblast activation and their subsequent release of Cxcl12. This chemokine subsequently attracts additional Cxcr4^+^ macrophages, thereby intensifying the cycle. The distinctive pathophysiology associated with silica exacerbates this cycle; its lysosomal toxicity triggers macrophage necroptosis or pyroptosis, leading to the release of damage‐associated molecular patterns, silica particles that perpetuate inflammation and facilitate the continuous recruitment of monocyte‐derived IMs.^35^, ^36^ Fibroblasts endeavor to encapsulate the particles; however, persistent Cxcl12/Cxcr4 signalling undermines these reparative processes. As a result, a significant alteration in macrophage populations was observed, characterised by a reduction in homeostatic AMs and an increase in recruited, pro‐fibrotic IMs. The therapeutic potential of disrupting this signalling axis is twofold. First, our findings corroborate that the Cxcl12/Cxcr4 interaction is a pivotal component of the fibrotic pathway, aligning with previous research indicating that its inhibition mitigates fibrosis through the suppression of TGF‐β.^14^ Second, we demonstrate that the Cxcr4 antagonist AMD3100 effectively disrupts this cycle. By inhibiting recruitment, AMD3100 decreases the density of pro‐fibrotic IMs, attenuates TGF‐β signalling, and weakens the crucial macrophage‒fibroblast interaction, ultimately reducing fibrosis. A recent study indicates that silica exposure induces RANKL‐dependent differentiation of pulmonary osteoclast‐like cells from monocytes and macrophages, which express TRAP and CTSK, thereby promoting fibrosis through sustained protease and hydrochloric acid release.^37^ Our findings elucidate a mechanistic model for the feed‐forward loop in which silica phagocytosis initiates the activation and pyroptosis of AMs. This process results in a population shift from homeostatic AMs to a substantial expansion of pro‐inflammatory, monocyte‐derived IMs, a population recently identified as phenotypically and functionally distinct.^38^ We further establish the pivotal role of these cells in fibrosis. They serve as a significant source of TGF‐β and, as demonstrated by Zhou et al., release matrix metalloproteinase 12, leading to endothelial dysfunction and matrix accumulation that perpetuates tissue injury. Therapeutically, targeting the Cxcl12/Cxcr4 axis with AMD3100 effectively disrupts this macrophage‐driven amplification loop, thereby reducing TGF‐β signalling, IM recruitment and fibrosis. This therapeutic strategy shows synergy with interventions targeting RANKL or Notch3, highlighting the potential of multi‐level approaches in addressing this complex disease.^37^, ^39^, ^40^

The pathological significance of the Cxcl12/Cxcr4 axis is definitively highlighted by our interventional studies. Administration of the Cxcr4 antagonist AMD3100 effectively disrupted the identified macrophage‒fibroblast circuit. AMD3100 demonstrated a dual mechanism of action: it directly inhibited the Cxcl12/Cxcr4 interaction and, crucially, suppressed Cxcl12 expression at its primary cellular source‐activated fibroblasts. AMD3100 mobilises haematopoietic stem cells by directly antagonising the Cxcl12/Cxcr4 axis. Recent evidence reveals an indirect effect: AMD3100 also reduces Cxcl12 expression in source cells. In lung fibrosis, activated fibroblasts overexpress Cxcl12 due to Twist1 loss and RelB activation; AMD3100 decreases this expression via intercellular feedback. Similarly, in acute lung injury, regulatory T cells lower epithelial Cxcl12 to mitigate fibrosis—an effect mimicked by AMD3100, suggesting immune modulation.^41^ Thus, AMD3100 exerts dual direct and indirect actions, with potential therapeutic implications beyond stem cell mobilisation.

We hypothesised that reduced Cxcr4 expression may limit macrophage activation and TGF‐β secretion. Lower TGF‐β suppressed fibroblast activation and Cxcl12 production, creating a positive feedback loop that amplifies inhibition of the fibrotic response to SiO_2_.^42^, ^43^ This combined effect led to a substantial reduction in the recruitment and activation of Cxcr4^+^ macrophages, along with a concomitant decrease in TGF‐β‐driven fibroblast activation and myofibroblast differentiation. Consequently, there was a significant attenuation of both fibrosis and the formation of silicotic nodules, which are pathologically characterised by dense subpleural fibrosis.^44^ Naturally, the molecular mechanisms governing interactions between fibroblasts and macrophages also encompass the CSF1‒CSF1R signalling pathway, thereby influencing disease onset and progression.^45^ The central role of this macrophage‒fibroblast amplification axis is reinforced by emerging literature on pro‐fibrotic macrophage subpopulations, not only in silicosis but also in other fibrotic contexts, such as the role of AXL^+^ monocytes in bleomycin‐induced lung injury.^34^, ^46^ Our findings define a critical pathogenic loop in silicosis: a TGF‐β/Cxcl12‐mediated macrophage‒fibroblast amplification axis, initiated by silica‐induced macrophage activation and sustained by Cxcr4‐driven recruitment. The efficacy of AMD3100 in disrupting this self‐perpetuating cycle highlights the Cxcl12/Cxcr4 axis as a promising therapeutic target for interrupting the progressive fibrotic cascade in this debilitating disease.

While we have demonstrated that fibroblast‐derived Cxcl12 facilitates the recruitment of pro‐fibrotic Cxcr4^+^ macrophages, the complete functional repertoire of these macrophages and the full spectrum of downstream pro‐fibrotic cascades they activate remain to be comprehensively elucidated. Additionally, the Cxcl12/Cxcr4 axis likely represents just one of several parallel pro‐fibrotic pathways. Fibrotic macrophages display high heterogeneity that extends beyond the conventional M1/M2 classification, driven largely by epigenetic and metabolic factors. This diversity gives rise to distinct subgroups, such as Spp1^+^ macrophages, which promote fibrogenesis, immune suppression and angiogenesis.^47^, ^48^, ^49^, ^50^ Within this regulatory network, Cxcr4 functions as a central node, mediating bidirectional crosstalk between macrophages and fibroblasts via the Cxcl12/Cxcr4/Pi3k/Akt axis. Under stimulation by TGF‐β, fibroblasts upregulate Cxcl12, which in turn induces M2 polarisation of macrophages. These polarised macrophages secrete additional TGF‐β, further driving myofibroblast differentiation. The Mif/Cxcr4 axis further links this loop to angiogenesis and metastasis. Thus, Cxcr4 serves as a key bridge connecting fibrosis, immunity and tumour progression, making it a promising therapeutic target.^51^ The Cxcl12/Cxcr4/Cxcr7 axis is actively being investigated for its roles in tumour progression and immune cell recruitment. Targeting this pathway has enabled combination therapy strategies using Cxcr4/Cxcr7 inhibitors alongside other anticancer agents.^52^ Beyond oncology, Cxcr4 antagonists also exhibit significant anti‐inflammatory effects, broadening their therapeutic potential. These findings support the rationale for developing drugs targeting the Cxcr4/Cxcl12 axis for both cancer and inflammatory diseases.^53^

AMD3100, an FDA‐approved Cxcr4 antagonist, shows novel anti‐fibrotic effects. Systemic use may disrupt haematopoietic stem cell homing, but local pulmonary delivery offers a safer alternative by increasing lung exposure while minimising systemic risks, warranting further preclinical and clinical validation.^54^ However, several limitations of this study should be acknowledged. First, due to technical cost constraints, the sample size was relatively small, which may limit the statistical power of the findings. Second, this study focused exclusively on the early stages of silicosis, without including observations from the late stages; consequently, whether AMD3100 exerts therapeutic effects in advanced disease remains unknown. Future research should prioritise: delineating the precise functional subpopulations within the Cxcr4^+^ macrophage pool, mapping the comprehensive interaction networks between macrophages and other structural and immune cells, and investigating combined therapeutic strategies that target this axis in conjunction with other key fibrotic pathways.

## 5 CONCLUSION

In summary, our multi‐omics analysis reveals a self‐perpetuating macrophage‒fibroblast circuit driven by the Cxcl12/Cxcr4 axis in silicosis. Disrupting this loop with AMD3100 significantly reduces fibrosis, positioning Cxcr4 as a promising therapeutic target for this disease.

## AUTHOR CONTRIBUTIONS

*Funding acquisition, methodology and writing—original draft*: Min Mu. *Data curation and writing*: Bing Li and Hangbing Cao. *Software*: Yuanjie Zou and Xiaolong Wu. *Methodology*: Ruiqing Yan and Zihao Xie. *Validation*: Fei Wang, Yehong Zhao and Miaomiao Du. *Conceptualisation*: Xinrong Tao. *Supervision and writing—review and editing*: Jianhua Wang.

## CONFLICT OF INTEREST STATEMENT

No potential conflict of interest was reported by the authors.

## ETHICS STATEMENT

This project has been approved by the committee on the ethics of Anhui University of Science and Technology Laboratory Animals (no. GZ2024‐070).

## Supporting information



Supporting Information

## Data Availability

The data produced in this investigation are not accessible to the public because they include confidential details belonging to the industry partner that financed and participated in the study. However, they can be obtained from the corresponding author upon a justified and reasonable inquiry.
