# Supplementary material for: Multi‐omics reveals a novel Cxcr4+ subpopulation of alveolar macrophages and therapeutic effect of AMD3100 in mice with advanced silicosis
Source: Clin Transl Med. 2026 May 27;16(6):e70705. doi: 10.1002/ctm2.70705 (PMC13240186; doi:10.1002/ctm2.70705)
Supplement: Supplementary file 1 — Supporting Information [file CTM2-16-e70705-s001.docx]

**SUPPLEMENTARY INFORMATION**

**
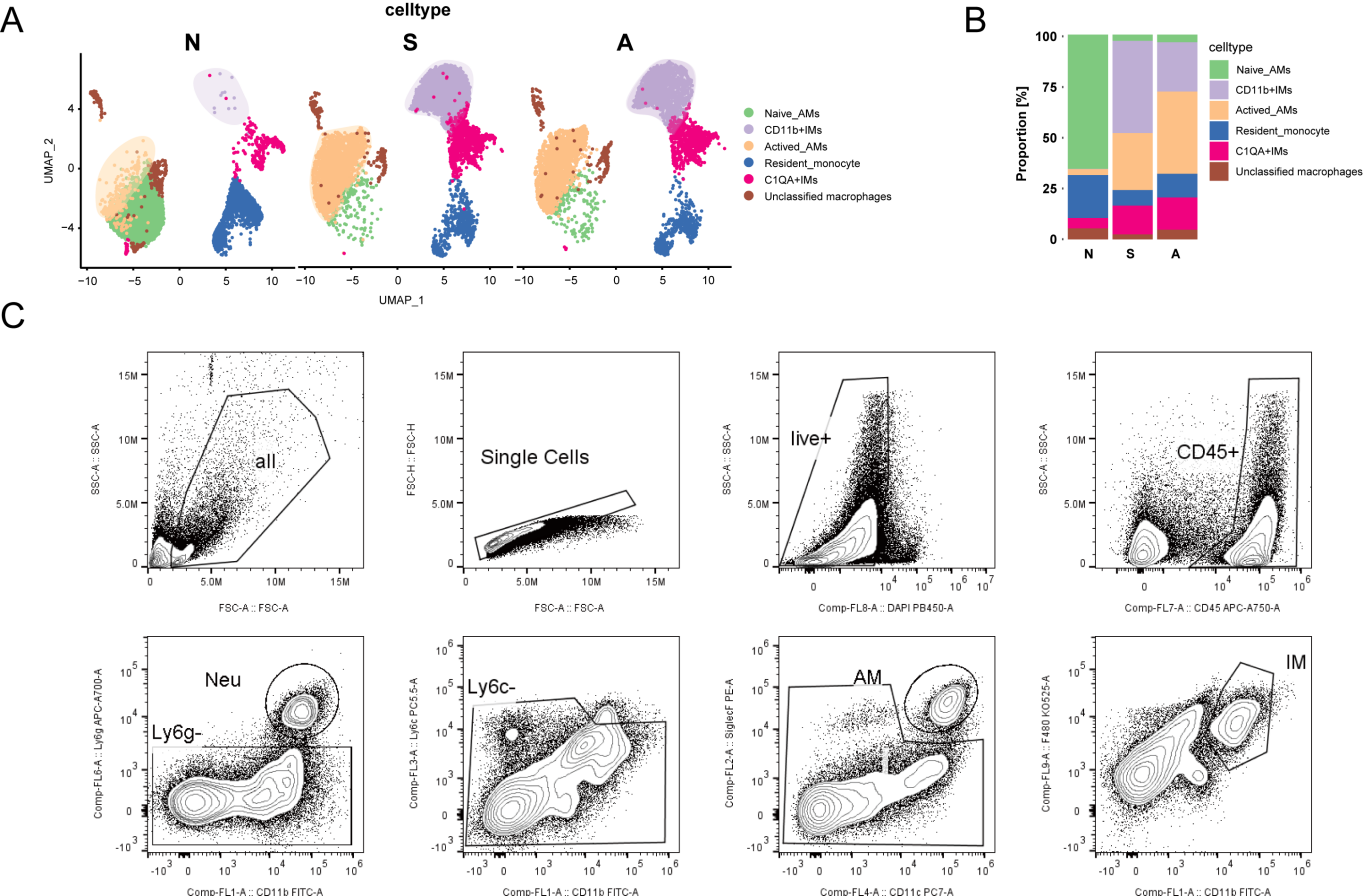
**

**FIGURE S1. Single-cell transcriptomic profiling and flow cytometric validation of macrophage subsets.**

1. The UMAP visualization of scRNA sequencing data delineates macrophage sub-clusters across different experimental groups.
2. The bar plot quantifies the proportion of each macrophage sub-cluster relative to the total macrophage population.
3. The flow cytometric gating strategy employed for identifying macrophage subsets (n = 3 per group).


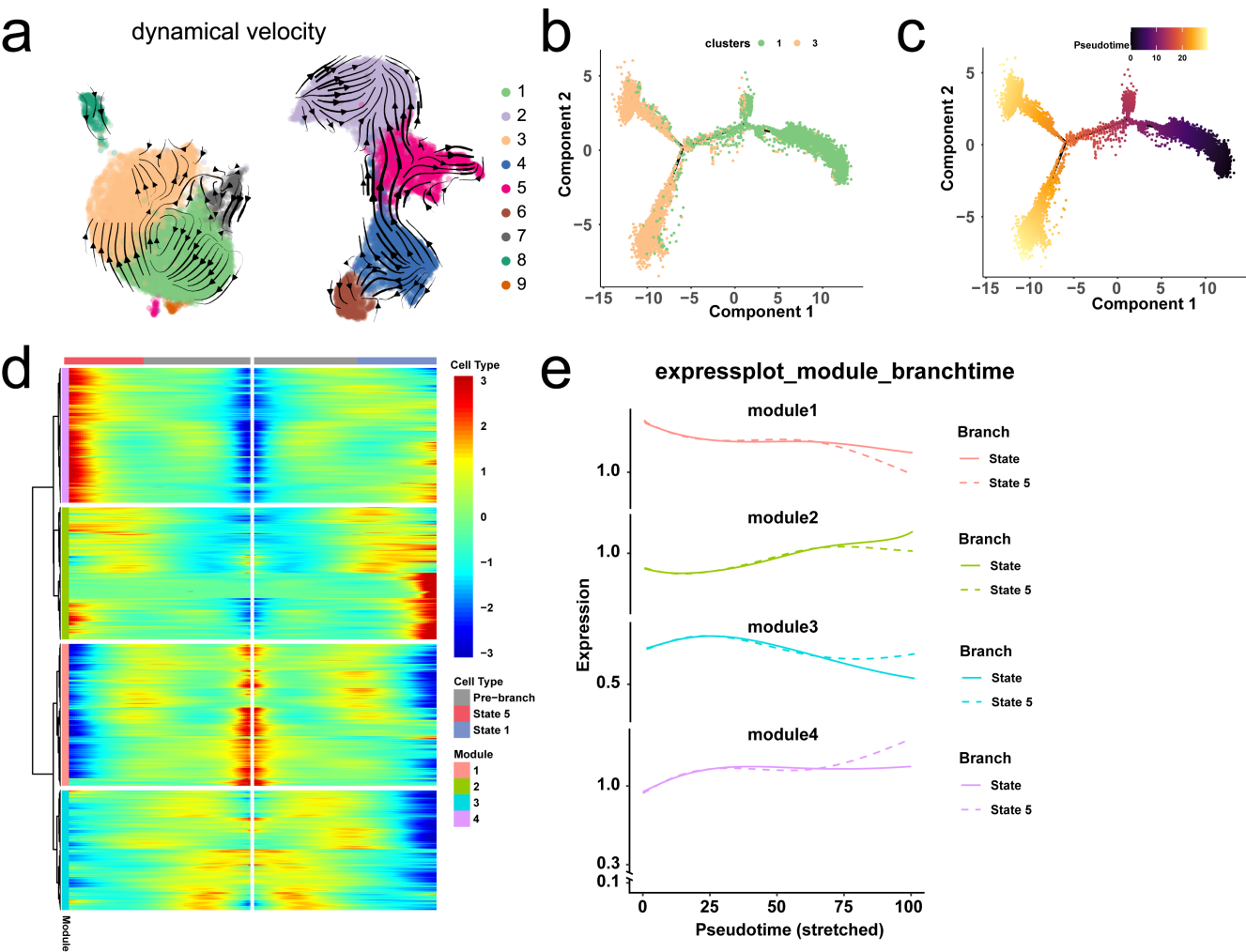


**FIGURE S2. Pseudo-temporal analysis of alveolar macrophages.**

1. The RNA velocity analysis of alveolar macrophages (AMs) reveals dynamic transcriptional transitions along the inferred trajectory, with arrows indicating the direction and magnitude of transcriptional flow.
2. C. The pseudo-temporal trajectory illustrates the evolutionary relationship between AM subclusters 1 and 3, suggesting a continuous differentiation process from Cluster 1 (homeostatic AMs) to Cluster 3 (activated/inflammatory AMs).
3. A heatmap of dynamically expressed genes, ordered by pseudo-time, highlights sequential transcriptional programs during macrophage activation.
4. An expression plot of key gene modules across pseudo-time and branch time demonstrates coordinated expression dynamics (n = 3 in each group).


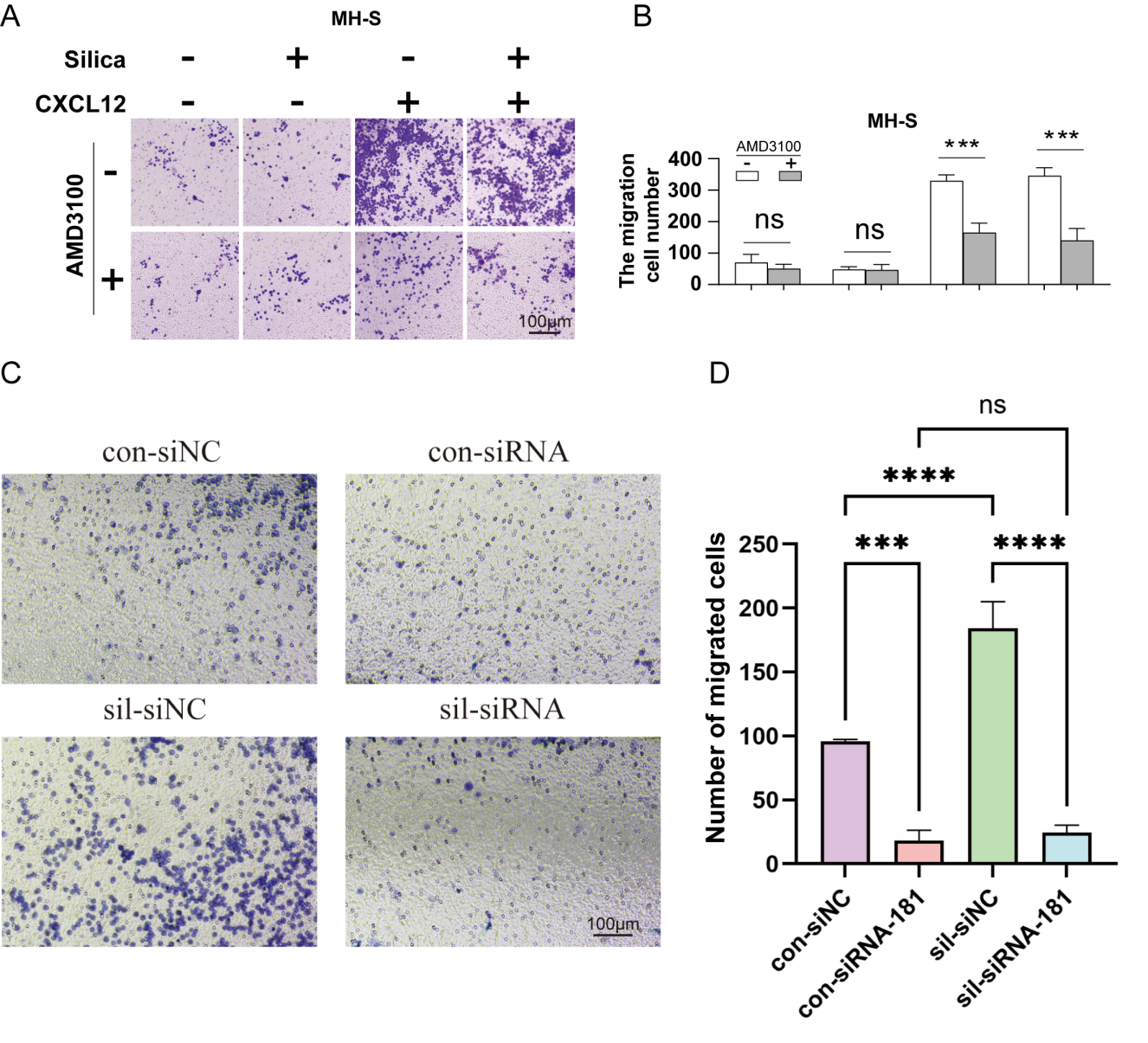


**FIGURE S3. Transwell assays of mouse alveolar macrophages and alveolar macrophages with fibroblasts following AMD3100 treatment.**

1. B. Migration and quantitative analysis of mouse alveolar macrophages (MH-S) cultured with fibroblast supernatant following treatment with SiO_2_ and AMD3100.

C-D. Migration and quantitative analysis of alveolar macrophages subjected to siRNA treatment prior to SiO_2_ exposure (n = 3 per group).


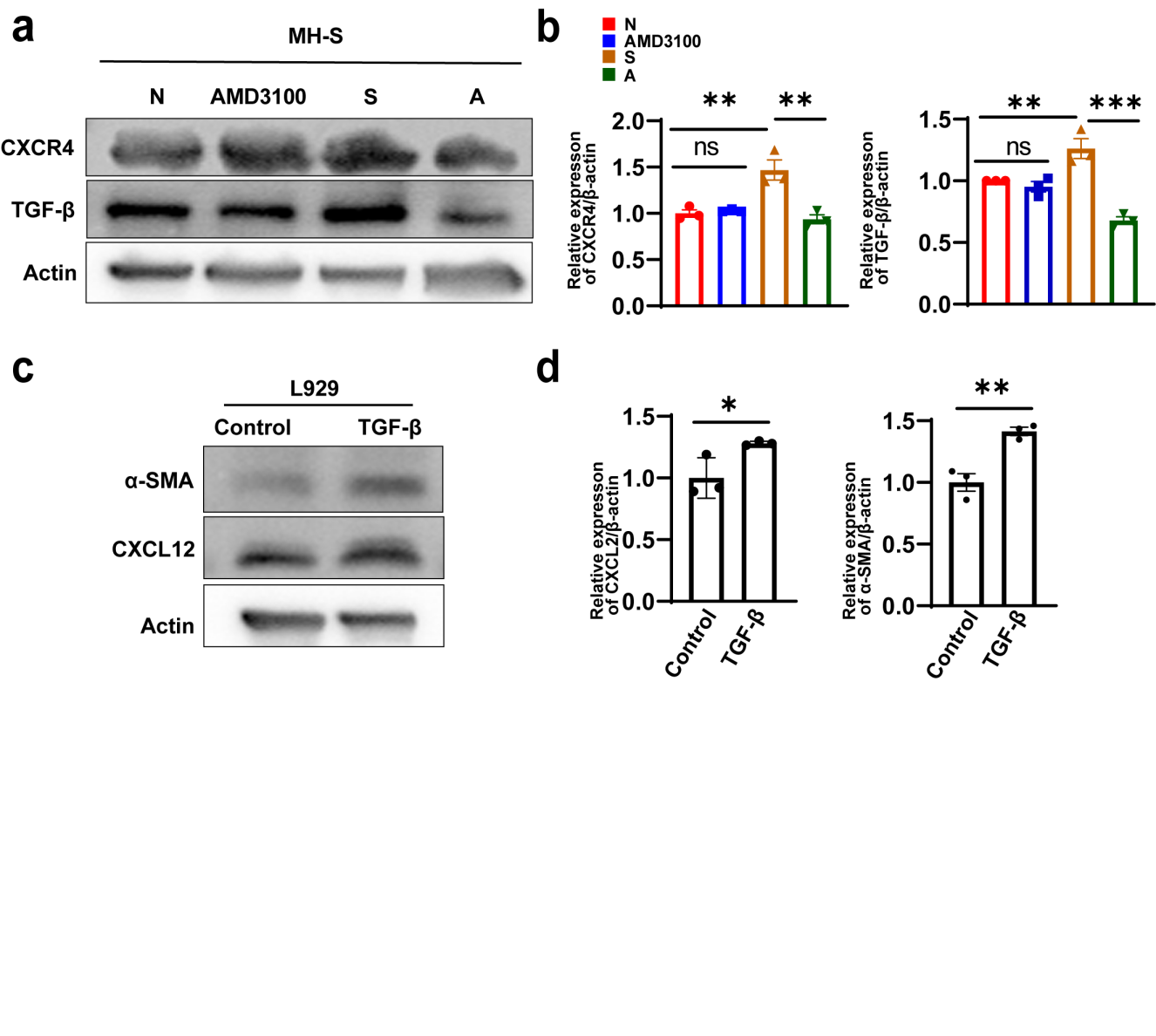


**FIGURE S4. Quantification of TGF-β and Cxcr4 expression levels in the alveolar macrophage cell line, as well as the expression of Cxcl12 and α-SMA in the L929 cell line following TGF-β treatment.**

1. B. The expression levels and quantification of TGF-β and Cxcr4 were analyzed in MH-S cells across different experimental groups.

C-D. Similarly, the expression levels and quantification of α-SMA and Cxcl12 were assessed in both control and TGFβ-treated L929 cell lines (Statistical analyses were performed by one-way ANOVA with student's t-test. *p < 0.05, **p < 0.01; n = 3 per group).
